# Supplementary material for: Characterization and Anti-Inflammatory Effects on Periodontal Ligament Cells of Citrus limon-Derived Exosome-like Nanovesicles Under Different Storage Temperatures
Source: Biomedicines. 2026 Jan 3;14(1):99. doi: 10.3390/biomedicines14010099 (PMC12838754; doi:10.3390/biomedicines14010099)
Supplement: Supplementary file 1 [file biomedicines-14-00099-s001.zip › biomedicines-4042826-supplementary.pdf]

Supplementary Materials

Characterization and Anti-Inflammatory Effects on Periodontal Ligament Cells of *Citrus limon*-Derived Exosome-like Nanovesicles Under Different Storage Temperatures

Yiming Ma, Chenhao Yu, Guojing Liu, Jia Liu * and Qingxian Luan *

Department of Periodontology, Peking University School and Hospital of Stomatology and National Center of Stomatology and National Clinical Research Center for Oral Diseases and National Engineering Laboratory for Digital and Material Technology of Stomatology and Beijing Key Laboratory of Digital Stomatology, Beijing 100081, China; 1810303111@pku.edu.cn (Y.M.)

* Correspondence: liujia_2632@bjmu.edu.cn (J.L.); kqluanqx@bjmu.edu.cn (Q.L.)

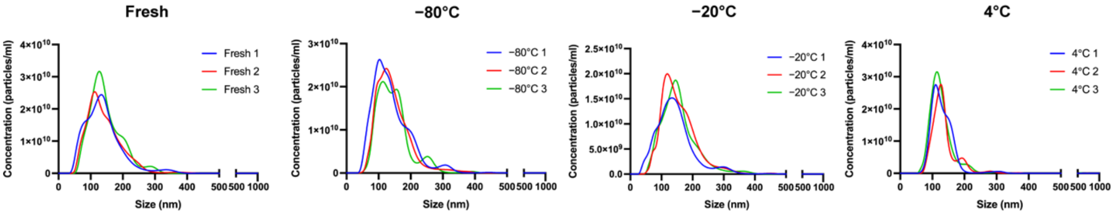

Figure S1. Technical replicates of NTA analysis (*n=3*).

To determine the half-maximal inhibitory concentration (IC_{50}), human periodontal ligament cells (hPDLCs) were treated with a gradient of fresh LELNs (0 - 100 μg/mL) alongside LPS stimulation. After 24 hours, total RNA was extracted, and the relative expression of TNF-α was quantified by qRT-PCR. The dose–response data were fitted to a four-parameter logistic model using GraphPad Prism to calculate the IC_{50} value and its 95% confidence interval. The percentage inhibition for each concentration was calculated as follows: Inhibition (%) = [1 – (Relative expression in LELNs-treated group) / (Relative expression in LPS-only group)] × 100%.

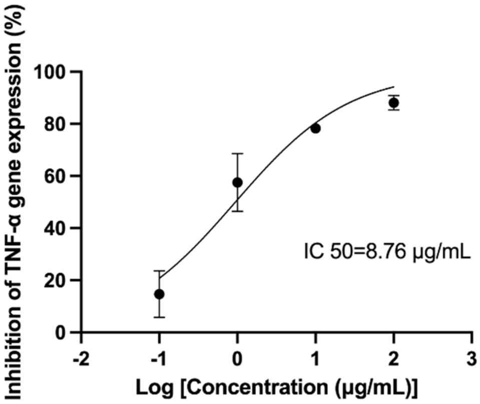

Figure S2. Dose-dependent inhibition of TNF-α gene expression by LELNs (*n=3*).

As shown in Figure S2, nonlinear regression analysis yielded an IC_{50} of 8.76 μg/mL (95% CI: 3.45 – 46.45 μg/mL). For the main experiments comparing the effects of different storage temperatures, LELNs from all groups were normalized to a final concentration of 1×10^9 particles/mL prior to cell treatment. This standardization was implemented to account for variations in particle recovery and protein content across storage conditions, thereby allowing for a direct comparison of the intrinsic bioactivity per particle.

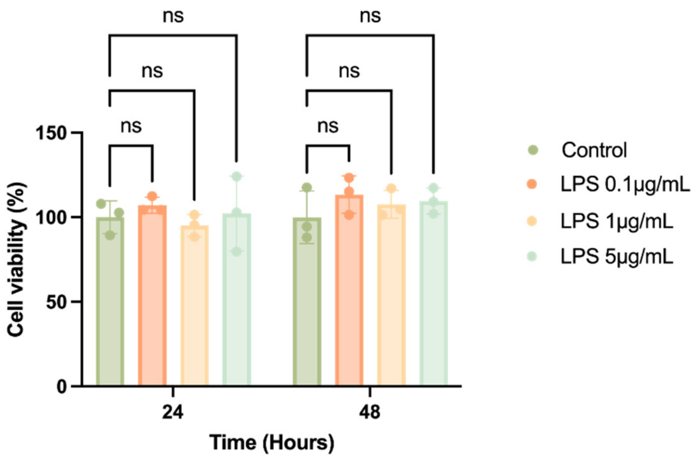

Figure S3. CCK-8 assessing the effect of LPS on cell viability across concentrations ranging from 0.1 μg/mL to 5 μg/mL, with incubation times of 24 and 48 h (*n*=*3*).
